# Risk factors threatening the sustainability of crop farming in South Africa

**DOI:** 10.4102/jamba.v17i1.1879

**Published:** 2025-10-16

**Authors:** Mulweli Matshidze, Vhuthu Ndou

**Affiliations:** 1 Department of Agronomy, Faculty of AgriSciences, University of Stellenbosch, Stellenbosch, South Africa; 2 Department of Radiochemistry, Necsa South African Nuclear Energy Corporation, Pelindaba, Pretoria, South Africa; 3 Department of Crop and Soil Science, Faculty of Agriculture, Oregon State University, Pendleton, United States of America

**Keywords:** energy, land reform, land degradation, pesticide contamination, sustainable agriculture

## Abstract

Agriculture is one of the main pillars of the economy in Southern Africa. According to the World Bank, the poverty rate in South Africa at the national level is 55%. Therefore, commercial farming is essential for economic development and poverty alleviation. However, certain factors pose a threat to the long-term viability of commercial crop farming. The aim of this review was to evaluate risk factors threatening commercial agriculture in South Africa. Several anthropogenic and environmental factors, such as climate change, pesticide contamination, land degradation, pests and diseases, were identified as risk factors threatening the sustainability of commercial crop farming in South Africa. Political factors such as the energy crisis and land reform were also identified as areas of great concern. A shift to regenerative agriculture is needed to ensure the sustainability of commercial agriculture because it is presently unsustainable.

**Contribution:**

Agriculture has a crucial role in South Africa’s economy, and the threats identified in this study need to be prioritised to help preserve farmers’ livelihoods and the overall economy by reducing financial risks, reducing unemployment and maintaining a consistent supply of agricultural exports.

## Introduction

Over the last six decades, food production has increased largely because of the intense use of inputs such as fertiliser, pesticides, irrigation, improved agricultural technologies (hybrid seeds) and expanding population (from approximately 4 billion in 1980 to 7 bn in 2024) (Food and Agriculture Organization [FAO] 2023). The increasing global population necessitates the continued large-scale production of food to satisfy demand (McDonald & Stukenbrock 2016). Commercial agriculture is the backbone of food production and is defined as large-scale production of crops and livestock for the market with the goal of making a profit (Mourya et al. 2025). The purpose of commercial agriculture is to generate profits from its operations and is considered a major earner of foreign exchange through exports of agricultural commodities (Department of Agriculture, Land Reform & Rural Development 2022). These enterprises exhibit a structured hierarchy. In contrast, subsistence farming aims to ensure food security for households rather than generating profit (Tibesigwa, Visser & Turpie 2016).

Commercial agriculture leads to the creation of artificial agricultural ecosystems that are primarily composed of monocultures. These agroecosystems (monocultures) lead to a decline in plant and animal diversity and encourage more virulent pathogens and arthropods compared to their wild ancestors because only specific high-yielding cultivars are grown. These make pathogen transmission easy and allow multi-infection by a different strain of the same pathogen (McDonald & Stukenbrock 2016). It has been reported that sugarcane, maize, wheat and rice are grown as monocultures and dominate global agriculture. Combined, they make up 1.9, 1.2, 0.8 and 0.8 bn tonnes of the total (9.6 bn tonnes) world production, respectively, and cover nearly 50% of the total global agricultural land (FAO 2023). Furthermore, the expansion of commercial agriculture has also resulted in increased greenhouse gas (GHG) emissions (Lenka et al. 2015).

In Africa, agriculture is one of the most important sectors because it provides employment, biofuel, raw materials for industry, economic contributions through exports, energy and food nourishment to rural and urban populations. Factors that hinder agricultural growth have undesirable consequences that threaten food security, poverty alleviation, economic growth and sustainable development (Tom-Dery et al. 2023). Most countries in Africa are developing (World Bank 2022), where hunger and poverty are common occurrences (Mugambiwa & Tirivangasi 2016). In South Africa, 814 518 people are employed in the agricultural sector and the related agricultural services industry (Statistics South Africa 2023). In addition, agriculture has already been dubbed as one of the sectors that will drive economic development. However, certain environmental factors threaten commercial agriculture, such as low rainfall (< 500 mm year^-1^), extreme temperatures, water pollution, biodiversity loss because of pests, soil degradation, etc. This is further exacerbated by the high evapotranspiration rate, which is greater than 1800 mm year^-1^. Furthermore, approximately 98 million hectares in South Africa are classified as semi-arid to arid (Du Preez & Van Huyssteen 2020; Hardy et al. 2011), and rainfall is limited both temporally and spatially (Du Preez & Van Huyssteen 2020).

The other issues threatening the sustainability of commercial farming include climate change and land degradation (Du Preez & Van Huyssteen 2020; Lenka 2015; Ma et al. 2021). Globally and at the national level, climate change is a major concern because commercial farming heavily depends on climate. According to the Intergovernmental Panel on Climate Change (IPCC), global yield losses of up to 50% should be expected in various major crops by 2050 (Zinyengere et al. 2014). Pesticides allow for timely pest control; however, pesticide resistance has also become a global issue worldwide (Ma et al. 2021). Climate change, in particular, has also increased the geographic range of many pests in previously pest-free areas. At the same time, pesticide use and resistance have increased in these areas (Ma et al. 2021). In this study, we review risk factors that threaten the sustainability of commercial farming in South Africa, with emphasis on crop farming.

### Ethical considerations

This article followed all ethical standards for research without direct contact with human or animal subjects.

## Discussion

### Gross domestic product, size, production and value of commercial agriculture in South Africa

In South Africa specifically, agriculture’s share of gross domestic product (GDP) was 2.2% in 2009 and 2.4% by the end of 2022 (Department of Agriculture, Land Reform & Rural Development 2022). In spite of its small share of total GDP, agriculture has significant upstream economic links, such as the production of seeds, fertilisers, pesticides and agricultural implements, and downstream economic links, such as supplying raw materials to the manufacturing industry and serving as a source of employment (Department of Agriculture, Land Reform & Rural Development 2022). In South Africa, smallholder farmers account for only 13% of total agricultural land, as compared to 87% of total agricultural land under commercial farming (Tibesigwa et al. 2016). Similarly, commercial farmers produce the highest agricultural outputs (Zantsi et al. 2025). The total number of registered commercial farms is 40 122, of which 64% are involved in crop production and mixed farming, whereas 40% are livestock production enterprises (Statistics South Africa 2017). For the year 2023, the total income earned by agriculture (field crops, animals and animal products) and allied services was R494.7 bn, which was an increase over the previous year’s (2021–2022) total of R450.2 bn; this represents a rise of 11.2% (Statistics South Africa 2023).

In South Africa, field crops such as maize, wheat and sugarcane are the major crops, and they make up the largest proportion of the cultivated land, depending on the area of production. Free State, Mpumalanga and North West provinces produced the most maize, 43.6%, 22.7% and 15.7% in 2017. Wheat is mostly produced in the Western Cape and Free State provinces, 38.4% and 27.8%, respectively. Sugarcane is commercially produced in KwaZulu-Natal and Mpumalanga provinces, but between 2007 and 2017, commercial production decreased from 15.7 m tonnes to 7.5 m tonnes. The major vegetables produced in South Africa include potatoes, onions, tomatoes and cabbage. The Free State province produced the most (36.8% of the total) potatoes, followed by Limpopo province (24.4%) and the Northern Cape province (10.4%) (Statistics South Africa 2017).

### Factors threatening the sustainability of commercial farming

#### Climate change

As per the IPCC, similar to other sub-Saharan African countries, South Africa is vulnerable to the negative impacts of climate change because of its substantial reliance on agriculture (Tibesigwa et al. 2016). Mugambiwa and Tirivangasi (2016) suggested that climate change could result in a 1.5% decrease in the South African GDP by 2050, and other allied industries will also be affected. As a result, studies have shown that climate change in South Africa may decrease crop yields. This is because South Africa may likely experience high temperatures and frequent but variable rainfall (Franke 2021; Ziervogel et al. 2014). Similarly, irrigation demand is expected to increase under climate change. South Africa is indeed a water-scarce country. Overall, climate change is expected to reduce the viticulture industry and have negative impacts on staples such as maize but minimal effects on the sugar industry (Ziervogel et al. 2014). An analysis by Tibesigwa et al. (2016) also showed that climate change will harm specialised crop farms, which are characteristic of commercial farming. Also, the increase in population in Africa and other trade partner countries in Asia will further exert pressure on food security in South Africa (Calzadilla et al. 2014). Climate change affects agricultural productivity by influencing water and/or precipitation, temperature and carbon dioxide (CO_2_) fertilisation. Therefore, these factors directly influence crop production (Calzadilla et al. 2014; Franke 2021).

#### Precipitation

Changes in rainfall patterns influence commercial irrigation but have a more direct effect on rainfed agriculture (Calzadilla et al. 2014). In irrigated agriculture, the major threat is groundwater availability, which will also be impacted by climate change and variability (Calzadilla et al. 2014). An analysis of 71 wheat cultivars across 17 South African locations using data from the Agricultural Research Council from 1998 to 2014 revealed that reduced precipitation and warming (+3 °C) would result in yield losses of up to 26% of rainfed winter wheat (Shew et al. 2020). For maize, it has been shown that a 1% decrease in rainfall will result in a 1% decrease in maize yield based on annual rainfall data from the nine provinces of South Africa between 1970 and 2006 (Blignaut, Ueckermann & Aronson 2009).

In the Free State, North West and Mpumalanga provinces, rainfall is expected to increase in the summer but not in the winter period. Rainfall will increase in December in the Free State from 85 to 90 and 97 mm, 56 to 60 and 65 mm in the North West province and 149 to 150 and 164 mm in Mpumalanga province for baseline (1990–2020), near future (2021–2050) and far future (2051–2080), respectively (Mangani et al. 2019). However, climate simulations of 1960 versus 2050 in three potato-producing regions (Western Cape, Free State and Limpopo provinces) revealed a decrease in rainfall in the Sandveld (Western Cape province) from 130.5 mm to 107.9 mm and 105.9 mm to 96.9 mm in Limpopo, except for the Free State, which will increase from 265 mm to 273 mm (Haverkort et al. 2013). Similarly, Franke, Muelelwa and Steyn (2020) studied the effects of climate change in various potato-producing regions and observed a decrease from 286 mm to 279 mm in Gauteng province, a decrease in Mpumalanga province from 305 to 297 and an increase in western Free State province from 234 mm to 262 mm between 1960 and 2050. Simulating rainfall projection also yielded uncertainty in rainfall patterns in the KwaZulu-Natal province sugarcane production area. Rainfall increased from 983 mm to 1071 mm under rainfed conditions and from 951 mm to 1034 mm under irrigated conditions using 1971–1990 as a baseline and 2046–2065 as the future prediction (Singels, Jones & Lumsden 2023).

Jones, Singels and Ruane (2015) simulated rainfall in three different sites of the sugarcane-producing sites of the KwaZulu-Natal province and reported that rainfall would increase from 998 mm to 1023 mm in one site (La Mercy) and decrease from 707 mm to 683 mm and 559 mm to 520 mm, respectively, in two sites (Malelane and Pongola) for the baseline (1980–2010) versus future scenario (2070–2100). Modelling the effect of climate change on the semi-arid climate area of KwaZulu-Natal province showed that the rainfall patterns will decline from 1151 mm in the past (1961–1991) to 1098 mm in the present (1995–2025) to 1010 mm in the future (2030–2060) under a high-emission scenario (CO_2_ > 1000 ppm) (Mabhaudhi et al. 2018). In rainy conditions, weather patterns will impact crop responses to inputs, including fertiliser, affecting the marginal value and optimal application levels. Growers may need to adapt their crops and production methods to continue growing crops. A decrease in total rainfall and increased variability can harm crop yields, thus affecting their economic viability and physical ability to grow (Olson 2013).

Lack of precipitation can also lead to drought, which is a major risk factor. Drought can be worsened by El Niño events (Baudoin et al. 2017). South Africa is highly prone to drought, and historic drought events include the 1982–1984, 1991–1992, 1994–1995 and 2015–2016 droughts. The major one is the 1991–1992 drought that resulted in 40% in crop losses and 49 000 job losses. Approximately 5m tonnes of cereal imports were needed to make up for all the yield losses (Baudoin et al. 2017). Drought forecasts and predictions will be necessary to minimise impacts. For proactive drought risk reduction and management, see Baudoin et al. (2017) for a full review.

#### Carbon dioxide

Walker and Schulze (2006) reported a one-tonne increase in maize grain yield as a result of elevated (two-fold) CO_2_ from 280 ppm to 560 ppm in South Africa. Similarly, a 2006 simulation of CO_2_ elevation (350–700 ppm) showed an increase in maize grain yield from 9 000 kg ha^-1^ – 10 000 kg ha^-1^ (baseline) to 11 000 kg ha^-1^ – 12 000 kg ha^-1^ based on data from 1971 to 2000 (Abraha & Savage 2006). For other crops, such as sugarcane, it has been reported that yield will increase from 120 000 (baseline) to 140 000 kg ha^-1^ in La Mercy, KwaZulu-Natal province, at an elevated CO_2_ of 734 ppm compared to 360 ppm (baseline) in 1980–2010 (baseline) versus 2070–2100 (future scenario) (Jones et al. 2015). Similarly, in the same province, it was reported that Bambara groundnut yield will also increase over time from 1 800 kg ha^-1^ presently (1995–2025) to 2 000 kg ha^-1^ (2030–2060) at elevated CO_2_ (> 1000 ppm) (Mabhaudhi et al. 2018). Simulating the impacts of elevated CO_2_ (> 1000 ppm; Representative Concentration Pathways (RCP) 8.5) on maize yields in the Eastern Cape province showed a decrease in maize yield from 11 310 kg ha^-1^ to 10 390 kg ha^-1^ mid-century (2040–2069) versus late century (2070–2099) (Choruma, Akamagwuna & Odume 2022). Based on the available CO_2_ studies, it does not appear as if elevated CO_2_ (+200 ppm) of the current (approximately 430 ppm) baseline will be a risk factor to commercial agriculture because elevated CO_2_ will increase crop yield, but warmer mean temperatures are likely to have the opposite effect (Ainsworth & Long 2020; Li et al. 2022).

#### Temperature

Models indicate a significant increase in temperature over South Africa, with projections showing a rise of 2 °C (1.5 °C – 2.5 °C) compared to a global rise of 1.5 °C (0.5 °C – 1.5 °C), especially in October and November (IPCC 2022). Under RCP 8.5, average temperature will increase from 18.09 °C in the 2020s and reach 22.2 °C by 2100 (end of the century) (Shayegh, Manoussi & Dasgupta 2020). Walker and Schulze (2006) reported a decrease in maize grain yield from 4051.5 kg ha^-1^ to 3421.8 kg ha^-1^ in conventional tillage systems as a result of elevated (+2 °C) temperatures in KwaZulu-Natal province. However, maize grain yield has been associated with an increase from approximately 9600 (baseline) to 11 233 kg ha^-1^ at +2 °C in mean air temperature, but a decrease from approximately 9600 (baseline) to 9000 kg ha^-1^ was observed at +4 °C in the midlands of KwaZulu-Natal province for the period 1971–2000 (Abraha & Savage 2006). As of 2019, Mangani et al. (2019) studied the potential impacts of extreme weather events and reported increased temperatures of approximately 15.9 to 16.8 and 17.3 °C in Bloemfontein, 16.4 to 17.1 and 17.9 °C in Lichtenburg and 15.71 to 16.3 and 16.9 °C in Mbombela for baseline (1990–2020), near future (2021–2050) and far future (2051–2080), respectively. This is significant because high temperatures and temperature extremes affect photosynthesis, crop development and plant reproduction (Rezaei et al. 2015). CRESE-maize modelling results by Li et al. (2022) showed that a +2 °C increase in temperature would also result in a decrease in yield for all the continents except Africa. This decrease in production would significantly affect the price of maize, thus creating a potential crisis in global food production. A summary of the effects of climate change on various crops in South Africa is shown in Table 1.

**TABLE 1:** Summary of the positive and negative effects of climate change on crop yield in South Africa.

Province	Crop	Model	Scenario	Impact on yield	Reason	Source
North West, Free State and Mpumalanga	Maize	CropSyst	RCP 4.5 and 8.5 (+3 °C – 4 °C)*	Decrease	Increased temperature in already warm provinces will result in shorter growth periods, less biomass accumulation and lower yields.	Mangani et al. 2019
Western Cape (Ceres), Free State (western) and KwaZulu-Natal	Potato	LINTUL	RCP 6.0 and RCP 8.5 (eCO_2_ 315 ppm – 550 ppm)	Increase	Most provinces studied showed high fresh tuber yields because of CO_2_ fertilisation in cooler years.	Franke et al. 2020
KwaZulu-Natal	Bambara groundnut	AquaCrop	A2 (eCO_2_ 218 ppm – 800 ppm)*	Increase	CO_2_ fertilisation resulting in an increase in photosynthesis.	Mabhaudhi et al. 2018
KwaZulu-Natal	Sugar cane	DSSAT-Cane growth	A2 (eCO_2_ 360 ppm – 734 ppm)	Increase	Faster canopy development because of increased radiation inception.	Jones et al. 2015
KwaZulu-Natal	Maize	CropSyst	+4 °C	Decrease	The 4 °C increase in temperature will shorten the growth season by 50 days, resulting in lower biomass accumulation and consequently low yields.	Abraha and Savage 2006
KwaZulu-Natal	Maize	CropSyst	eCO_2_ (280 ppm – 560 ppm)	Increase	Because of a 10% increase in rainfall and CO_2_ fertilisation.	Walker and Schulze 2006
Western Cape, Free State and Limpopo	Potato	LINTUL	eCO_2_ (315 ppm – 550 ppm)	Increase	CO_2_ fertilisation resulting in increased photosynthetic rates and tuber yields.	Haverkort et al. 2013
Eastern Cape	Maize	EPIC	RCP 4.5 and 8.5 (+3 °C – 4 °C)*	Decrease	Increased temperature resulting in short growth periods, less biomass accumulation and lower yields.	Choruma et al. 2022

Note: Please see the full reference list of this article, Matshidze, M. & Ndou, V., 2025, ‘Risk factors threatening the sustainability of crop farming in South Africa’, *Jàmbá: Journal of Disaster Risk Studies* 17(1), a1879. https://doi.org/10.4102/jamba.v17i1.1879, for more information. Updated definitions of the RCP-Representative Concentration Pathways and climate scenarios (A2, B1) are available on the IPCC (https://www.ipcc.ch/). eCO_2_: elevated carbon dioxide.

CropSyst, Crop Systems Simulation Model; LINTUL, Light Interception and Utilisation; AquaCrop, Water Productivity Model for Crop Production; DSSAT, Decision Support System for Agrotechnology Transfer; EPIC, Environmental Policy Integrated Climate model; RCP, Representative Concentration Pathways; CO_2_, Carbon dioxide.

* , approximated from the Intergovernmental Panel on Climate Change (IPCC) scenarios.

#### Land degradation

In South Africa, there is also evidence of the transformation of natural landscapes for agricultural activities, which results in land degradation and a decrease in water quality. This has been the case in the Bot River in the Western Cape province (De Waal, Miller & Van Niekerk 2022). In addition, freshwater pollution in South Africa because of commercial farming activities has been well documented (De Waal et al. 2022). The Vaal River is no exception. The Vaal River is the third-longest river in South Africa and supplies various irrigation schemes, mines and industries in the Gauteng province. However, evidence of anthropogenic activity, including agricultural activity such as ploughing in riparian zones and high use of fertiliser and pesticides, has resulted in nutrient loading in the Vaal River, which can be potentially harmful to humans and aquatic life. Phosphate, ammonium ions and nitrate ions common in many agricultural fertilisers were found in eutrophic levels, which has resulted in deterioration in Vaal River water quality (Ntshalintshali 2019).

In the Eastern Cape province, there is evidence of an increase in agricultural cropland abandonment. Using geographic mapping techniques, it was revealed that approximately 37% of cropland in the Macubeni catchment was abandoned, which has increased the risk of erosion because 20% of those lands were significantly degraded (Sibiya, Clifford-Holmes & Gambiza 2023). Soil degradation has also been assessed on undisturbed, versus grazed and cultivated land in the North West province. It was found that the grazed and cultivated soil was severely compacted relative to undisturbed land (Materechera 2014). Boardman et al. (2017) reported desertification in the Sneeuberg uplands of the Eastern Cape province because of sheep overgrazing. This is no surprise because the major land usage in South Africa is dedicated to grazing, cropland and forestry (83%, 12% and 0.8%, respectively). These former practices result in serious land degradation (Van Huyssteen & Du Preez 2023). Table 2 shows evidence of land degradation in South Africa because of agricultural activity. Some peer-reviewed studies on land degradation in South Africa are limited to communal and smallholder farmers and not necessarily to commercial enterprises.

**TABLE 2:** Selected studies that show evidence of land degradation in South Africa.

Province	Agricultural activity	Type	Effect	Indicator	Source
KwaZulu-Natal	Cattle path and grazing	Soil (chemical degradation)	Decrease	Soil organic carbon and nitrogen	Abdalla et al. 2024
Limpopo	Overgrazing and erosion	Biological	Decrease	Vegetation cover	Kgaphola et al. 2023
Eastern Cape	Abandoned cop land	Soil (chemical degradation)	Increase	Erosion	Sibiya et al. 2023
KwaZulu-Natal	Burning for sugarcane harvesting	Soil (chemical degradation)	Decrease	Organic matter	Dominy, Haynes and Van Antwerpen 2002
KwaZulu-Natal,	Excessive use of agrochemicals	Land	-	Eutrophication	Shabalala, Combrinck and McCrindle 2013
Limpopo	Overgrazing	Soil (chemical degradation)	Increased	Erosion	Nzuza et al. 2022
Eastern Cape	Overgrazing and other natural factors	Land and soil	Increased	Desertification, Gully, etc.	Boardman et al. 2017
Western Cape	Agrochemicals	Land	Increase	Nitrogen	De Waal et al. 2022
North West	Grazing and cultivation	Soil (chemical degradation)	Decrease	Organic matter and microbial biomass	Materechera 2014

Note: Please see the full reference list of this article, Matshidze, M. & Ndou, V., 2025, ‘Risk factors threatening the sustainability of crop farming in South Africa’, *Jàmbá: Journal of Disaster Risk Studies* 17(1), a1879. https://doi.org/10.4102/jamba.v17i1.1879, for more information.

#### Pests and diseases

In South Africa, there are various pests and diseases and pathogens that threaten crop production, such as fall armyworm (*Spodoptera frugiperda*) and *Fusarium* spp. (Makgoba et al. 2020; Van Rensburg et al. 2014). *Fusarium* species not only reduces crop yields but also releases mycotoxins in grains, which, when consumed, have carcinogenic effects. *Fusarium* spp. was detected in maize grain from the North West, Northern Cape and Free State provinces in 2014 and caused significant interest internationally (van Rensburg et al. 2014). However, fall armyworm is a significant maize pest, and 57% of smallholder farmers in the Capricorn District, Limpopo province, reported reduced crop yield because of fall armyworm infestations (Makgoba et al. 2020). Effective management strategies, including biological and cultural methods to control this pest, have been reviewed by Kasoma, Shimelis and Laing (2020). Furthermore, more than 30 arthropods in South Africa have been reported to be resistant to at least one pesticide. Examples include the South African bont tick (*Amblyomma hebraeum*) and onion thrips (*Thrips tabaci*) (both of which can serve as disease vectors). This evidence is based on data from the Arthropod Pesticide Resistance Database (APRD) (APRD 2025). Other important pests in South Africa include the brown locust, African migratory locust, red locust and the southern African desert locust. These are four major locust species that have historically caused plagues and significant economic damage to agricultural crops (Price 2023). Outbreak frequency, patterns and control strategies for some of these locusts were also reviewed by Price (2023).

#### Pesticide contamination

Pesticides play a major role in controlling pests; this results in increased crop yields and thus increasing profits. However, there is a consensus that pesticides are being used injudiciously, particularly in developing countries, resulting in their unsustainability. The non-judicious use has resulted in the degradation of microbial communities (Meena et al. 2020). Furthermore, besides causing environmental contamination, pesticides negatively impact non-target organisms, such as beneficial insects and wildlife, thereby leading to a decline in their populations (Horak, Horn & Pieters 2021). In South Africa most studies have focused on the presence of pesticides in the environment, such as in rivers, and their potential contamination of human beings (Table 3). An example of such studies includes a study by Rimayi et al. (2018), where they monitored water quality in Hartbeespoort Dam and 10 nearby sites (inlets and outlets) that feed the dam. Using mass spectrometry analysis, the results revealed triazine herbicides in the Hartbeespoort Dam and terbuthylazine in all 10 sampling sites. Because these pesticides have an effect on endocrine and immune systems, this poses a serious risk to humans and wildlife. Because of the increased reports of organochlorine pesticides in aquatic wildlife, a study was initiated to determine if pesticides were present in African catfish (*Clarias gariepinus*). The fish was sampled in the Klip River catchment that drains the Witwatersrand in Gauteng, South Africa. The results showed the presence of organochlorides in catfish tissue; some were because of historic use, and some were more recent exposure, which may have carcinogenic risk because African catfish is a valuable food source in South Africa (Pheiffer et al. 2018). The presence of these pesticides in the environment impedes South Africa from achieving agricultural sustainability (Horak et al. 2021).

**TABLE 3:** Selected studies that studied pesticide contamination in the environment in South Africa.

Province sampled	Pesticide detected	Sample	Species or associated process	Source
Gauteng	Dichlorodiphenyltrichloroethane	River	Detected in aquatic species	Pheiffer et al. 2018
Western and Eastern Cape, Gauteng, KwaZulu-Natal and Free State	Atrazine and terbuthylazine	Drinking water	-	Odendaal et al. 2015
Gauteng, North West	Organochlorines	Various	Detected in aquatic species (*Clarias gariepinus*)	Barnhoorn et al. 2015
North West	Atrazine and terbuthylazine	Dam	Detected in aquatic species	Rimayi et al. 2018
Western Cape	Various insecticides, fungicides and herbicides	Air, Soil	-	Degrendele et al. 2022

Note: Please see the full reference list of this article, Matshidze, M. & Ndou, V., 2025, ‘Risk factors threatening the sustainability of crop farming in South Africa’, *Jàmbá: Journal of Disaster Risk Studies* 17(1), a1879. https://doi.org/10.4102/jamba.v17i1.1879, for more information.

#### Energy

**FIGURE 1:**
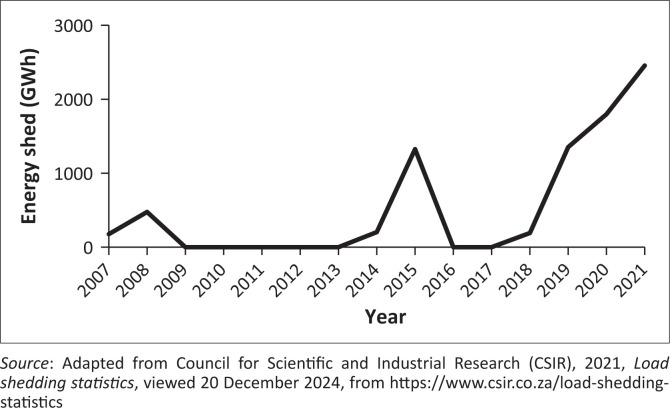
Cumulative power outages in South Africa (redrawn from data from the Council for Scientific and Industrial Research [CSIR]).

For energy generation to be possible, there has to be a balance between energy generation and supply. In South Africa, the utility company known as the Electricity Supply Commission (Eskom) implemented power cuts because of a higher demand for electricity than can be supplied. This is informally referred to as load shedding. Under load shedding, the load or demand for electricity is forcibly reduced to maintain the said balance (Booysen, Van Der Berg & Van Der Walt 2023). Cumulative power outage in South Africa is shown in Figure 1. Existing literature shows power cuts are affecting the cold chain, which in turn threatens food quality and shelf life, especially for agricultural products that have high perishability and thus ultimately influences the selling price (Cloete, Pienaar & Van Der Merwe 2023; Esterhuizen 2022). Irrigation, fertigation and other water management practices in orchards have also been negatively affected. Some commercial enterprises have resorted to generators, but this is unsustainable because of the associated fuel costs. Although load shedding by definition is a planned outage, there is evidence of unplanned power disruptions that are negatively affecting livestock production facilities that require power to simulate special environments for animal production, such as the poultry sector. The other concern is abrupt load-shedding notices that do not allow growers enough time to purchase fuel and make other provisions. There have also been reports of growers incurring additional costs because of the extra hours they have to pay farmworkers to make up for lost time and damage to equipment because of power outages (Esterhuizen 2022). Although load shedding started in South Africa in 2007 with only 120 h shed (equivalent to 5 days), the 2020s saw an increase in load shedding. Therefore, there will probably be more studies that will quantify agricultural losses on a national level as a result of load-shedding; however, the impact of load shedding has been estimated to be billions of dollars, and this obviously is a major risk factor threatening commercial farming enterprises in South Africa (Esterhuizen 2022).

#### Land reform

Lastly, the issue of land reform is also worth mentioning because there is a relationship between land reform, commercial farming and food production. In the period 1980–2000, Zimbabwe implemented the Fast Track Land Reform Programme (FTLRP). In the 1980s, Zimbabwe was a net maize exporter, and by 2000, the country could no longer feed itself (Dabale & Chiringa 2014; Hove & Gwiza 2012). In South Africa, the Department of Agriculture, Land Reform, and Rural Development (DALRRD) also has similar land reform policy programmes, such as the Proactive Land Acquisition Strategy (PLAS) and South Africa’s State Land Lease and Disposal Policy (SLLDP). According to Sebola and Tsheola (2014), certain aspects of land reform, such as the manner of land claimants and the manner of land use by the benefactors, may threaten the profitability and sustainability of commercial agriculture. An example is when land claims are made on economically active commercial farms for residential purposes or resettlement purposes, which may halt agricultural productivity and economic viability. However, based on the current available peer-reviewed literature, there is not enough evidence to show that land reform may completely restrict commercial farming in South Africa because only 10% of South Africa’s agricultural land has been redistributed since 1994 (Zantsi et al. 2025). Although equity, economic growth, rural development and food security are the goals that land reform aims to accomplish (Kirsten et al. 2016), some experts disagree, stating that those aims have not been met and land reform has just opened doors for a select few elites (from the previously oppressed group) to access land through the aforementioned SLLDP policies; for a full review, see Mtero, Gumede and Ramantsima (2023). Furthermore, Sebola and Tsheola (2014) and Kirsten et al. (2016) have suggested that land reform has failed to fulfil its objectives of wealth distribution, economic growth and historical redress. This is supported by rampant unemployment, poverty and unemployment in South Africa, as reported by the World Bank and Statistics South Africa.

## Conclusion

Agriculture is a crucial sector in the South African economy, making it essential to mitigate the main climate and political risks to the sector. Certain risk factors can be managed by various strategies, for example pests and diseases can be managed by sound pest and disease management practices. Unfortunately, the other risk factors covered in this analysis, such as climate change, land degradation, energy and land reform, may require cooperation between the government and private sectors to develop comprehensive regulatory frameworks and incentives to invest in renewable energy. Investing in renewable energy in South Africa could help not only with the energy crisis but could also play a role in reducing climate change by reducing overreliance on fossil fuel use. Similarly, pesticide contamination mitigation and, thus, protection of aquatic and environmental ecosystems will also require collaboration among researchers, management entities and government agencies to monitor pesticides so that they do not reach non-target areas. On the farm level, sustainable and regenerative agriculture can play a role in restoration and conservation.
